# Identification of novel macrolides with antibacterial, anti-inflammatory and type I and III IFN-augmenting activity in airway epithelium

**DOI:** 10.1093/jac/dkw222

**Published:** 2016-07-25

**Authors:** James D. Porter, Jennifer Watson, Lee R. Roberts, Simren K. Gill, Helen Groves, Jaideep Dhariwal, Mark H. Almond, Ernie Wong, Ross P. Walton, Lyn H. Jones, John Tregoning, Iain Kilty, Sebastian L. Johnston, Michael R. Edwards

**Affiliations:** 1 Airway Disease Infection Section, National Heart Lung Institute, Imperial College London, London, UK; 2 MRC & Asthma UK Centre for Allergic Mechanisms of Asthma, London, UK; 3 Pfizer Inc., Cambridge, MA, USA; 4 Mucosal Infection and Immunity Group, Section of Virology, Imperial College London, London, UK

## Abstract

**Background:**

Exacerbations of asthma and COPD are triggered by rhinoviruses. Uncontrolled inflammatory pathways, pathogenic bacterial burden and impaired antiviral immunity are thought to be important factors in disease severity and duration. Macrolides including azithromycin are often used to treat the above diseases, but exhibit variable levels of efficacy. Inhaled corticosteroids are also readily used in treatment, but may lack specificity. Ideally, new treatment alternatives should suppress unwanted inflammation, but spare beneficial antiviral immunity.

**Methods:**

In the present study, we screened 225 novel macrolides and tested them for enhanced antiviral activity against rhinovirus, as well as anti-inflammatory activity and activity against Gram-positive and Gram-negative bacteria. Primary bronchial epithelial cells were grown from 10 asthmatic individuals and the effects of macrolides on rhinovirus replication were also examined. Another 30 structurally similar macrolides were also examined.

**Results:**

The oleandomycin derivative Mac5, compared with azithromycin, showed superior induction (up to 5-fold, EC_50_ = 5–11 μM) of rhinovirus-induced type I IFNβ, type III IFNλ1 and type III IFNλ2/3 mRNA and the IFN-stimulated genes viperin and MxA, yet had no effect on IL-6 and IL-8 mRNA. Mac5 also suppressed rhinovirus replication at 48 h, proving antiviral activity. Mac5 showed antibacterial activity against Gram-positive *Streptococcus pneumoniae*; however, it did not have any antibacterial properties compared with azithromycin when used against Gram-negative *Escherichia coli* (as a model organism) and also the respiratory pathogens *Pseudomonas aeruginosa* and non-typeable *Haemophilus influenzae*. Further non-toxic Mac5 derivatives were identified with various anti-inflammatory, antiviral and antibacterial activities.

**Conclusions:**

The data support the idea that macrolides have antiviral properties through a mechanism that is yet to be ascertained. We also provide evidence that macrolides can be developed with anti-inflammatory, antibacterial and antiviral activity and show surprising versatility depending on the clinical need.

## Introduction

Type I and type III IFNs are antiviral cytokines made by a wide range of cells in response to virus infection and ligation of various pattern recognition receptors (PRRs). Type I IFNαs and IFNβ signal via the IFNα receptor (IFNAR)-1 and IFNAR2 complex, while type III IFNλs (also designated IL-29, IL-28A and IL-28B in humans) act via the IL-10Rβ and IFNλ1Rα chains.^1^ Type I and type III IFN production are one of the first initiators of the host's innate immunity against virus infection^2^ and induces the ‘antiviral’ state. IFNs are known to initiate the expression of >300 different genes termed IFN-inducible genes (ISGs), via signalling through the type I and type III IFN signalling complexes,^3–6^ and many of the protein products of these ISGs have been shown to be directly antiviral in a range of models.^7^ ISGs can be extracellular, such as chemokines or cytokines that attract other cells of the immune system, or IFNs themselves with a classical example being IFNβ inducing IFNα and IFNλs.^8^ Most ISGs, however, are intracellular proteins that act to prevent virus protein trafficking, virus RNA synthesis or virion assembly and release. Viperin and MxA are two examples of intracellular antiviral ISGs.^9,10^ Viperin is thought to act on viral protein and also lipid trafficking within the host cell,^11,12^ whilst MxA is a small GTPase that localizes to the endoplasmic reticulum and inhibits viral replication.^13^ Several specific ISGs have been shown to be directly antiviral in models of respiratory virus infection, including influenza virus,^14^ respiratory syncytial virus and human rhinovirus (RV).^15^

Studies by Wark and colleagues in 2005 and later by Contoli *et al.* in 2006 initially described deficiencies in the ability of asthmatic primary human bronchial epithelial cells (BECs) to produce type I and type III IFNs in response to RV infection.^16,17^ Subsequent studies have confirmed the role for deficient IFN production in asthmatic BECs cultured from both children and adults.^18–20^ Studies have also been extended to other cell types including airway macrophages^16,21^ and peripheral blood-derived dendritic cells.^22,23^ Impaired type I and type III IFN production have also been observed in cells derived from patients with COPD.^24^ This growing body of evidence suggests that augmenting deficient IFN production in response to RV infection in asthmatic and COPD patients could be a novel target for therapeutic intervention in the context of RV-associated asthma and COPD exacerbations.

Currently, there are no therapies that attempt to restore deficient IFN levels in the treatment of asthma; however, recently, inhaled recombinant IFNβ therapy has been used in a Phase II clinical trial examining naturally occurring infections in a cohort of mild–moderate asthmatics.^25^ Encouragingly, in a subset of patients, IFNβ therapy reduced symptoms and improved lung function recovery time post-infection compared with placebo. Apart from respiratory diseases, IFN therapy is currently a mainstay in the treatment of viral hepatitis, used in combination with the antiviral agent ribavirin.^26^ Increases in host IFN production have been shown to directly translate into antiviral activity in a clinical context.^27^ Due to the expense associated with using recombinant IFNs as biological interventions in human disease, one alternative approach is small molecule agonists of IFNs or their associated antiviral pathways that illicit the same IFN-mediated protective effect.

The aim of this study was to identify one or more novel macrolides with antiviral-augmenting ability that had similar or greater potency or efficacy to that of azithromycin. The antiviral effects of azithromycin shown in previous studies in healthy BECs and cystic fibrosis (CF) BECs were both modest and variable, producing up to 200%–500% augmentation of type I and type III IFN induction following RV infection, but with limited potency *in vitro.*^28,29^ We therefore embarked on a screen of 225 novel macrolides in an attempt to identify compounds with stronger effects on innate host responses to RV in terms of both efficacy and potency. We thus report the identification of a novel macrolide, Mac5, that could augment primary BEC gene expression of RV-induced IFNβ and IFNλs and the ISGs MxA and viperin. Mac5 was also able to decrease RV replication in BECs. This augmentation of antiviral activity was up to 5-fold superior to that seen with azithromycin treatment. Mac5, however, showed no inhibition of *Escherichia coli*, *Pseudomonas aeruginosa* or non-typeable *Haemophilus influenzae* (NTHi) growth, but did suppress the growth of *Streptococcus pneumoniae*. We then further assessed 30 novel analogues of Mac5 and identified further macrolides with similar antiviral efficacy, but with improved anti-inflammatory and/or antibacterial properties. Importantly, the data overall show that antiviral, IFN-mediated pathways can be targeted independently of inflammation and that macrolides represent an exciting and versatile class of drug for the treatment of exacerbations of chronic airway diseases.

## Materials and methods

### Macrolides

Azithromycin (Sigma–Aldrich, USA), 225 novel macrolides and 30 further macrolides based around the Mac5 structure (Pfizer UK) were obtained as dry powders and made up in 70% EtOH. Azithromycin (Sigma–Aldrich) and all novel macrolide compounds (Pfizer, USA) were prepared as 0.1 M solutions by adding the appropriate amount of 70% EtOH to the macrolide, vortexing for ≥10 s and sonicating in a water bath for up to 3 min to ensure complete dissolution. Stock solutions were kept at −20°C.

### Cells and viruses

All cell cultures were maintained in a humidified 37°C incubator with 5% CO_2_ (New Brunswick, USA). BECs were maintained in fully supplemented BEC growth medium (BEGM) (Lonza). Culture medium was aspirated and replaced with fresh medium every 48 h. HeLa cells were maintained in DMEM supplemented with 10% heat-inactivated FBS (PAA, UK), 2% 1 M HEPES (Gibco, USA) and 1% NaHCO_3_ (Gibco). BEAS-2B cells were maintained in RPMI medium supplemented with 10% heat-inactivated FBS, 2% 1 M HEPES and 1% NaHCO_3_. Major group RV16 and minor group RV1b were cultured in HeLa cells as previously described^30^ and identity confirmed using serotype-specific antibodies and titration in HeLa cells. The RV1b stock was *Mycoplasma*-free and stored at −80°C.

### Primary BEC culture from commercial sources

Human BECs (Lonza) were split according to the supplier's recommended protocol. When cells reached ∼90% confluency, the culture medium was removed and the cell monolayer rinsed twice with 4 mL of HEPES-buffered saline solution per flask (Lonza); the final rinse was completely aspirated to ensure total removal of cell culture medium. Next, 2 mL of trypsin/EDTA (Lonza) was added to each flask and flasks were agitated by hand to ensure complete coverage of the cell monolayer. Flasks were then incubated at 37°C (5% CO_2_) for ∼3 min. Flasks were tapped by hand firmly and detached cells were removed and added directly to 4 mL of trypsin-neutralizing solution (TNS; Lonza) per flask. A further 1 mL of trypsin/EDTA was added to flasks to remove stubbornly adherent cells and returned to the incubator for 1 min, after which each flask was thoroughly rinsed with 4 mL of TNS and the cell solution pooled. The cell solution was then centrifuged at 1200 rpm for 6 min and the supernatant discarded. The cells were thoroughly resuspended in 10 mL of fully supplemented BEGM; this solution was then split between fresh 75 cm^2^ flasks and the total volume was made up to 10 mL per flask. Routinely, two flasks (from thawing) were split into nine and grown to 90% confluency. For experiments, three of those flasks were split into nine more, while cells from the remaining six flasks were counted and seeded onto culture plates at a density of 0.5 × 10^5^ cells/mL in fully supplemented BEGM for all experiments. Cells were not grown beyond six passages.

### Culture of asthmatic BECs from bronchial brushings

Bronchial brushings were obtained via bronchoscopy of atopic, poorly controlled asthmatics as previously described.^31^ Cells were dislodged and grown until confluency and passaged as previously described.^20,31^ All experiments were performed with cells at p2, as described below.

### Treatment of cells with azithromycin and novel macrolides

Working concentrations of each macrolide (100–0.78 μM) were generated by thoroughly thawing the stock solutions in a 37°C water bath for 10 min, vortexing for 10 s and diluting the stock into the appropriate volume of serum/supplement-free medium. Working solutions were then vortexed and added to cell culture wells. To normalize for effects of vehicle, a volume of 70% EtOH diluted in medium equal to that of the top dose of macrolide solution in each experiment was used to dilute the other solutions and was also added onto cells and included as a ‘vehicle control’. All treatments were for 24 h prior to RV infection. In certain experiments, cell viability after 24 h of macrolide treatment was assessed by an MTT assay (Sigma–Aldrich) according to the manufacturer's recommended protocol.

### Infection of BEAS-2B cells and BECs with RVs

Prior to infection, all cells were placed in infection medium (BECs in supplement-free bronchial epithelial basal medium and BEAS-2Bs in RPMI with 2% FBS) for 24 h. Stock RV1b was diluted in the relevant infection medium prior to application to cell cultures to give an approximate moi of 1. RV was then added (200 μL per well of a 12-well plate or 50 μL for a 48-well plate) and cultures were incubated at room temperature for 1 h with agitation. Virus was then removed and the cultures were washed three times with infection medium. Cultures were placed in infection medium and incubated for a specified amount of time at 37°C.

### Transfection of BEAS-2B cells and reporter screening assay

Superfect (Qiagen) was used for all transfections, according to the manufacturer's recommended protocol. Transfections were performed in 48-well culture plates (Nunc). Stock plasmids were at 1 μg/mL and stored at −80°C. An initial master mix of reporter plasmid (IFNβ-Luc or IFNλ1-Luc grown in-house, 0.2 μg/well) was diluted in serum-free RPMI an internal control vector (Renilla, 0.05 μg/well) making a total of 0.25 μg/well DNA.

### Reporter assay

Relative light units (RLU) were measured using a luminometer with Promega's Dual Luciferase kit (Promega, USA) according to the manufacturer's instructions. Relative promoter activity was calculated by dividing the RLU of the reporter plasmid (IFNβ-Luc or IFNλ1-Luc) by the RLU of the internal control vector (Renilla). To assess any augmentation of promoter activity, relative promoter activity was then expressed as a percentage of RV-induced promoter activity (RV induction being 100%).

### RNA extraction, cDNA synthesis and quantitative PCR

Supernatants from cell cultures were removed and 350 μL of RLT (Qiagen) buffer was added to each culture well. Cells were lysed and stored at −80°C. All RNA extractions were performed using RNeasy (Qiagen) kits according to the manufacturer's instructions. RNA was eluted by adding 42 μL of RNA-free H_2_O and centrifuging at 14 000 rpm for 1 min. RNA was converted into cDNA using an Omniscript RT kit (Qiagen) according to the manufacturer's instructions, in a total reaction volume of 60 μL. Each mixture was incubated at 37°C for 60 min and frozen at −80°C until used. Quantification of mRNA copy numbers was measured by TaqMan PCR (7500 Fast Real-Time PCR System; Applied Biosystems, USA). Each PCR mixture contained 6.25 μL of QuantiTect Probe PCR Master Mix (Qiagen), sense and antisense primers and an optimized ratio 175 nM probe and 1 μL of unknown or standard cDNA. Concentrations of primers and sequences of primers and probes are as previously reported.^28^ The quantitative PCR cycling program was as follows: 50°C for 2 min, 94°C for 10 min followed by amplification cycles of 94°C for 15 s and 60°C for 15 s to a total of 45 cycles. An eight-point standard curve was used to determine mRNA copy number in unknown samples. Standards were diluted in nuclease-free H_2_O to the top standard of 10^7^ copies/μL; sequential 1 : 10 dilutions were produced to 10^0^ copies/μL.

### ELISA

ELISA was used to quantify IL-6 and IFNλ1 (IL-29) from cell culture supernatants using the manufacturer's recommended protocol (R&D Systems, USA). Absorbance at 450 nm was measured using a SpectraMax Plus (Molecular Devices, USA) ELISA plate reader and absorbance levels were determined by extrapolation of a four-parameter standard curve generated in SoftMax Pro software (Molecular Devices). Detection limits for the assays were: IL-6, 15 pg/mL; IL-8, 15 pg/mL; and IFNλ1, 30 pg/mL.

### Antibacterial assay using E. coli culture

Initially, *E. coli* (Invitrogen, UK) from frozen glycerol stocks was plated out using aseptic techniques onto LB agar plates and incubated at 37°C for 24 h. An individual colony was picked and grown in liquid LB overnight at 37°C with agitation to allow the bacteria to reach log-phase growth. From this starter culture, a 1 : 100 dilution was made into fresh medium containing macrolides or vehicle control and incubated for up to 12 h at 37°C with agitation. Sequential sampling of cultures was performed at specific timepoints and analysed by spectrometry at OD_600_.

### Antibacterial assay using respiratory pathogens

*S. pneumoniae* strain D39 was grown from a glycerol stock (1 : 200 dilution) for 16 h with shaking (150 rpm) at 37°C with 5% CO_2_ in the presence or absence or each macrolide (50 μM) or vehicle. Single colonies of *P. aeruginosa* strain PAO1 were resuspended in LB broth and grown overnight at 37°C with shaking at 200 rpm. Overnight cultures were diluted 1 : 100 in fresh LB broth in 96-well culture plates and macrolides (50 μM) or vehicle added. Plates were incubated at 37°C with shaking at 200 rpm for 4 h. Each macrolide was tested in triplicate. NTHi strain R2866 was grown as a starter culture overnight at 37°C in 5% CO_2_ with shaking at 200 rpm. This was then diluted 1 : 100 in fresh BHI broth in the presence or absence of each macrolide or vehicle control for 4 h. Sampling of each bacterial culture was performed at the specified timepoints and analysed by spectrometry at OD_600_.

### Statistical analysis

For data pertaining to gene or protein expression by cultured normal BECs, the mean and SEM were displayed in all graphs. Gene and protein expression data were normalized to percentage over induction by positive control (where the positive control was expressed as 100%). As the data were not normally distributed, a one-way Kruskal–Wallis non-parametric analysis with Dunn's comparison was used to assess significance between groups. Data from time course experiments were analysed by two-way analysis of variance (ANOVA) with Bonferroni's post-test. Data from dose–response assays were analysed using non-linear regression on a sigmoidal dose–response curve to generate EC_50_ and *R*^2^ values. Two-tailed Spearman correlation analysis was employed where stated. For bacterial growth curves, the AUC was calculated and analysed by ANOVA with Bonferroni's post-test. All statistical analysis employed a CI of 95%. *P* values of <0.05 were considered statistically significant.

## Results

### Optimization of a screening assay using IFN promoter reporter constructs to identify antiviral properties of novel macrolides

We first used the human IFNβ promoter (Figure S1, available as Supplementary data at *JAC* Online) as a reporter gene to screen 225 novel macrolides. Our aim was to identify novel macrolides with IFN-augmenting properties greater than azithromycin and so we decided on a three-phase screening programme (summarized in Figure S1 and Table S1). Phase 1 was a quick screen (*n* = 1) using reporter assays to determine the highest responders compared with both RV1b infection alone and also with RV1b infection with azithromycin (positive control). Phase 2 was further confirmation of these hits by three repeats with the IFNβ promoter and also the IFNλ1 promoter reporter assays, which is activated by a similar mechanism to IFNβ.^8,32,33^ Phase 3 was the final validation of the selected hits, by several experiments in primary BECs and assessment of effects on endogenous RV-induced IFN and ISG mRNA expression.

### Screening assays revealed 15 novel macrolides with IFN-augmenting activity

**Figure 1.**
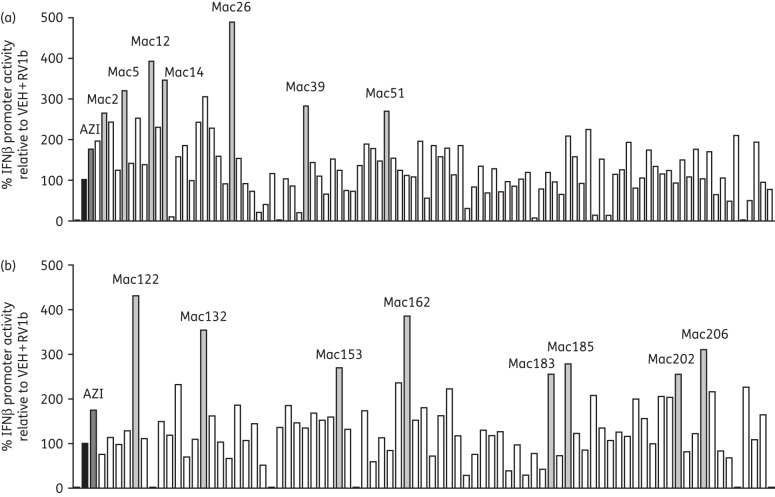
Novel macrolides differentially augmented RV1b-induced IFNβ promoter activity in BEAS-2B cells. (a) BEAS-2B cell cultures were treated with 50 μM of novel macrolide compounds (1–115) for 24 h prior to RV1b infection for 24 h. Seven compounds (light grey bars) augmented IFNβ promoter activity 1.5-fold above the level of azithromycin (dark grey bar). Compounds that were toxic at 50 μM were excluded. (b) BEAS-2B cell cultures were treated with 50 μM of novel macrolide compounds (116–225) for 24 h prior to RV1b infection for 24 h. Eight compounds (light grey bars) augmented IFNβ promoter activity 1.5-fold above the level of azithromycin (dark grey bar). Compounds that were toxic at 50 μM were excluded. Data expressed as percentage of RV1b + vehicle (VEH; black bars; 100%). *n* = 1.

**Figure 2.**
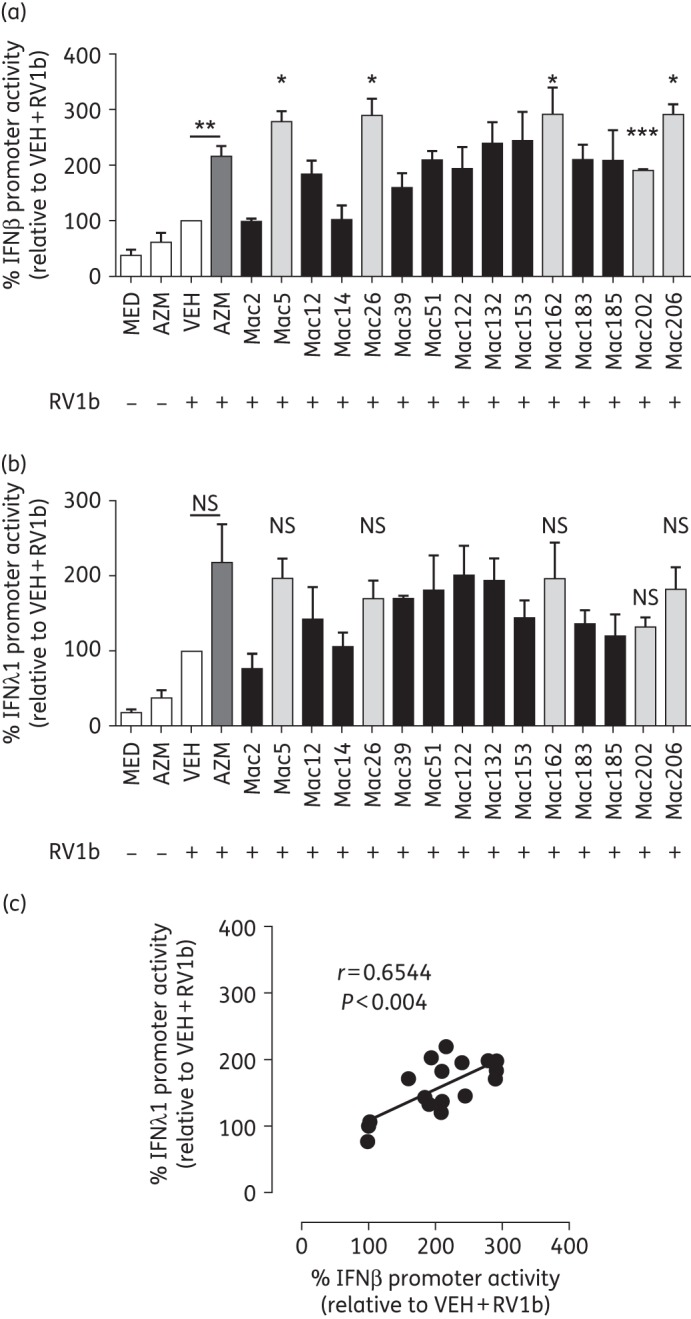
Novel macrolides augmented RV1b-induced IFNβ and IFNλ1 promoter activity. (a) Five novel macrolides significantly augmented RV1b-induced IFNβ promoter activity in BEAS-2B cell cultures. (b) Five novel macrolides showed trends for augmenting IFNλ1 activity, although none to a significant level. (c) BEAS-2B cell cultures treated with novel macrolides and subsequently infected with RV1b exhibited a strong correlation between levels of IFNβ promoter activity and IFNλ1 promoter activity. Data expressed as percentage change of RV1b + vehicle (VEH) induction. *P < 0.05, **P < 0.01 and ***P < 0.001 versus RV1b + VEH. *n* = 3. Correlations assessed by Spearman correlation. NS, not significant.

Results of phase 1 of the screening process are shown in Figure 1. A number of novel macrolides gave induction of the RV1b-induced IFNβ reporter equal to or greater than that of azithromycin, while some macrolides completely suppressed IFNβ promoter induction and were not studied further. Some compounds were toxic at the 50 μM dose used and were reassessed at 10 μM and found to have no effect on RV1b-induced IFNβ promoter induction (data not shown). Compounds that were toxic at both 50 and 10 μM were not studied further. Figure 1 shows there were 15 macrolides that had ≥1.5-fold-induction+3×SD better than that seen with azithromycin and were progressed into the next phase of screening. The results of the second phase are shown in Figure 2. The top 15 hits were repeated three times in both the IFNβ promoter and the IFNλ1 promoter assay. The data show that Mac5, Mac26, Mac162, Mac202 and Mac206 all produced approximately >200% induction of RV1b-induced promoter activation, which was equal to or greater than seen with azithromycin and RV1b. These five also showed significant induction versus RV1b and vehicle for IFNβ (Figure 2a) and showed similar, but non-significant, induction for the IFNλ1 promoter (Figure 2b). The induction of the IFNβ and IFNλ1 promoters was similar for all 15 hits and induction of both reporters for all 15 hits correlated positively (Figure 2c).

### The 15 novel macrolides with IFN-augmenting activity are derivatives of azithromycin, erythromycin and oleandomycin

The 15 novel macrolides identified in the IFN assays belonged to three classes of macrolides. Mac26, Mac132, Mac153 and Mac183 were derivatives of azithromycin, Mac162, Mac202, Mac206, Mac39, Mac122 and Mac185 were derivatives of erythromycin and Mac2, Mac5, Mac12, Mac14 and Mac51 were derivatives of oleandomycin. How each class of macrolide compared during phase 2 of the screening is shown in Figure S2. While azithromycin and erythromycin derivatives gave more robust inductions of IFNβ, IFNλ1 and combined IFNβ and IFNλ1 promoter induction, there was no significant difference between each of the three classes.

### Structural description of the top five selected macrolides

**Figure 3.**
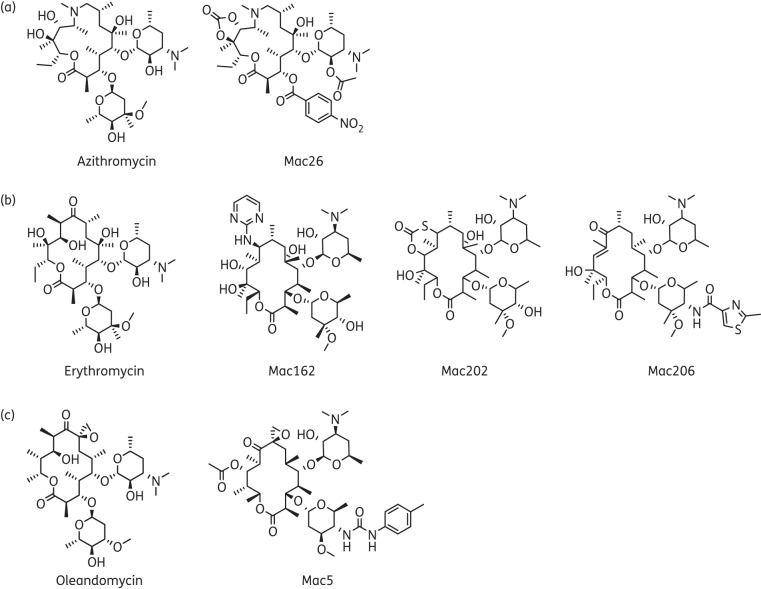
Structures of the five novel derivatives of azithromycin, erythromycin and oleandomycin selected from screening. (a) Azithromycin and its derivative Mac26. (b) Erythromycin and its derivatives Mac162, Mac202 and Mac206. (c) Oleandomycin and its derivative Mac5.

Structures of the top five selected macrolides are shown in Figure 3. Stereochemistry has been included where possible. Cellular toxicity studies showed that each macrolide was not toxic by MTT assay (Figure S3). They belong to three classes of macrolides: azithromycin, erythromycin and oleandomycin. For comparison, the parental compounds are also shown. Mac26 was found to be an azithromycin analogue (Figure 3a), Mac162, Mac202 and Mac206 are erythromycin analogues (Figure 3b) and Mac5 is an oleandomycin analogue (Figure 3c). Azithromycin is a 15-membered macrocyclic ring containing a methyl-substituted nitrogen atom. Mac26 differs from the parental azithromycin in three ways: the two hydroxyl groups at positions C12 and C13 are cyclized into a carbonate; the cladinose sugar has been replaced by a nitrobenzoyl group; and on the desosamine sugar, the hydroxyl at position C2 has been converted into an acetate. Erythromycin is another 14-membered macrolide very similar to azithromycin, but lacks the nitrogen atom in the macrocyclic ring. The three erythromycin derivatives identified in the screen show extensive modifications from the parental compound. Mac162 exhibited a substitution of the ketone moiety for an aminopyrimidine at C9 of the macrocyclic ring. Mac202 contains a cyclic thiocarbonate group in place of the C9 ketone. Mac206 possesses an unsaturated ketone and a 2-methyl-4-amidothiazole substituent on the cladinose sugar. Oleandomycin is a 14-membered macrolide with the two sugars oleandrose and desosamine. Mac5 differs from oleandomycin by the acetate at C11 of the 14-membered macrocyclic ring and the addition of a tolyl urea at position C4 of the oleandrose ring.

### Oleandomycin derivative Mac5 induced endogenous type I and type III IFN and ISG transcription, but not pro-inflammatory cytokines, in primary BECs

**Figure 4.**
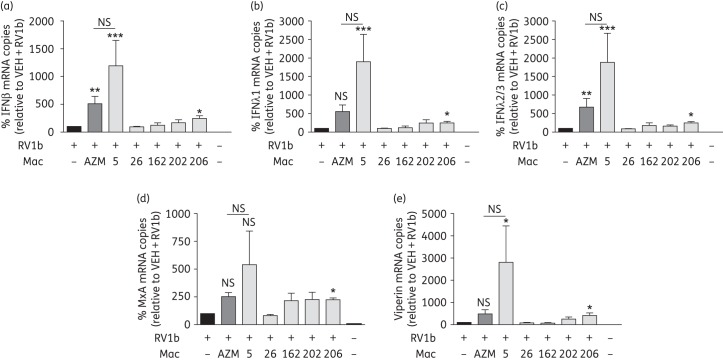
Novel macrolides augmented mRNA levels of RV1b-induced IFNs and ISGs in primary BECs. (a) Mac5 and Mac206 augmented RV-induced IFNβ in normal BEC cultures. Mac26, Mac162 and Mac202 failed to significantly augment mRNA levels of IFNβ. (b) Mac5 and Mac206 augmented IFNλ1 mRNA in normal BEC cultures. Mac26 and Mac162 failed to augment mRNA levels of IFNλ1. Mac202 showed trends to augment IFNλ1 levels above that of RV1b alone. (c) Mac5 and Mac206 significantly augmented RV-induced IFNλ2/3 mRNA in normal BEC cultures. Mac26, Mac162 and Mac202 failed to significantly augment mRNA levels of IFNλ2/3. (d) Mac206 augmented RV-induced MxA in normal BEC cultures. Mac5, Mac162 and Mac202 all showed a trend for augmenting RV-induced MxA. Mac26 failed to augment MxA levels. (e) Azithromycin, Mac5 and Mac206 augmented RV1b-induced expression of viperin. Mac202 showed trends for augmenting viperin mRNA; however, this was not statistically significant. Mac26 and Mac162 failed to augment. All data expressed as percentage change of RV1b + vehicle (VEH; black bars) induction. *P < 0.05, **P < 0.01 and ***P < 0.001 versus RV1b + VEH. *n* = 8. NS, not significant.

**Figure 5.**
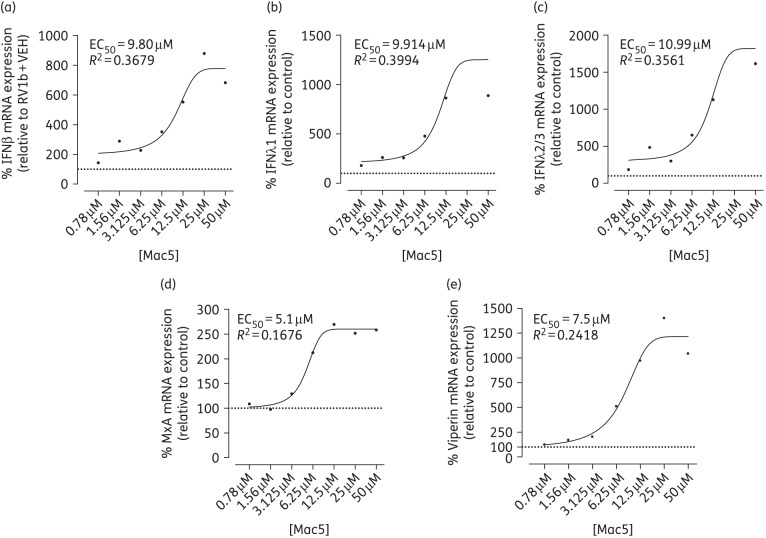
Mac5 showed a dose-dependent augmentation of type I and type III IFN mRNA in normal BECs. (a) Mac5 was titrated 1 : 2 and showed a sigmoidal dose–response to augmenting RV1b-induced IFNβ mRNA at 24 h post-infection. (b) Mac5 was titrated 1 : 2 and showed a sigmoidal dose–response to augmenting RV1b-induced IFNλ1 mRNA at 24 h post-infection. (c) Mac5 was titrated 1 : 2 and showed a sigmoidal dose–response to augmenting RV1b-induced IFNλ2/3 mRNA at 24 h post-infection. (d) Mac5 was titrated 1 : 2 and showed a sigmoidal dose–response to augmenting RV1b-induced MxA mRNA at 24 h post-Mac was titrated 1 : 2 and showed a sigmoidal dose–response to augmenting RV1b-induced viperin mRNA at 24 h post-infection. Data expressed as percentage change of RV1b + vehicle (VEH) (100% indicated by horizontal broken line). *n* = 3.

As we identified five novel macrolides that could augment RV-induced IFNβ and IFNλ1 promoter activation in a cell line, we next sought to confirm these findings with endogenous gene expression using commercially available, primary BECs (phase 3). We found that of all five macrolides, Mac5 was the only consistent performer significantly augmenting IFNβ, IFNλ1, IFNλ2/3 and viperin, while showing increased trends of induction compared with vehicle-treated RV1b-infected controls for MxA mRNA (Figure 4). The mean augmentation of IFN or ISG mRNA was ∼1000% compared with vehicle-treated RV1b-infected cells, but had a wide range (500%–2800%). No other macrolide gave significant induction versus vehicle-treated RV1b-infected cells and no macrolide including Mac5 induced IFN of ISGs spontaneously in the absence of infection (Figure 4). Furthermore, comparisons with azithromycin-treated RV1b-infected cells in the same experiments showed that Mac5 gave higher levels of induction for all mRNAs measured; however, none was significantly greater than levels of mRNA induction compared with azithromycin (Figure 4). At best, Mac5 was ∼5-fold better at augmenting RV-induced viperin mRNA compared with azithromycin, but was mostly ∼2–3-fold more effective than azithromycin for most genes at the same dose. We also checked induction of MxA and viperin protein by western blotting for each macrolide with RV1b infection (Figure S4) and found that Mac5 robustly induced viperin protein and gave a modest induction of MxA relative to RV1b-infected cells. Mac26 and Mac206 also showed induction of viperin protein relative to RV1b. Consistent with the mRNA data, no macrolide in the absence of infection induced MxA or viperin protein. Further studies showed that Mac5 gave sigmoidal dose–response curves and augmented RV-induced IFN and ISGs in a dose-dependent manner with EC_50_s ranging from 5 to 11 μM (Figure 5). In comparison, the dose–response curves of azithromycin for the same genes were not sigmoidal, indicating a different relationship between dose and effect studied (Figure S5). A complete comparison between Mac5 and azithromycin is shown in Table S2. Mac5 also did not significantly augment RV-induced IL-6 or IL-8 mRNA (Figure S6) or affect the basal transcription of these genes (data not shown).

### Mac5 reduced RV1B replication in primary healthy BECs and asthmatic BECs

**Figure 6.**
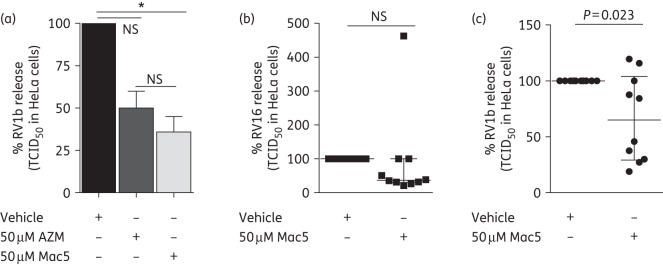
Mac5 inhibited RV1b replication in BECs. (a) Mac5 (light grey), but not azithromycin (dark grey), significantly inhibited RV1b replication in commercially available BEC cultures after 48 h. (b) Mac5 showed trends for reducing RV16 release in asthmatic BECs, which were not significant versus vehicle-treated cells (black). (c) Mac5 significantly reduced RV1b release in asthmatic BECs, which were not significant versus vehicle-treated cells. Data expressed as percentage change of RV1b + vehicle (100%) induction. *P < 0.05 versus RV1b + vehicle. *n* = 4 commercially available normal BECs and *n* = 10 asthmatic BECs. NS, not significant.

We next assessed the ability of Mac5 to reduce RV replication (Figure 6), reasoning that a robust IFN response would translate into reductions in virus load at later timepoints. We found that in commercially available, healthy normal BECs, Mac5 significantly reduced RV1b load at 48 h post-infection by ∼64% (Figure 6a), but was not significantly better than azithromycin, which showed a modest, non-significant reduction of RV release of ∼50%. We also assessed the effects of Mac5 in BECs derived from adult poorly controlled asthmatics (clinical details presented in Table S3). Mac5 did not significantly reduce RV16 (Figure 6b), but significantly reduced RV1b (Figure 6c) replication at 48 h in the asthmatic BECs. Overall, 7/10 subjects showed decreases in both RV16 and RV1b release compared with vehicle-treated cells.

### Mac5 possessed limited antibacterial activity

**Figure 7.**
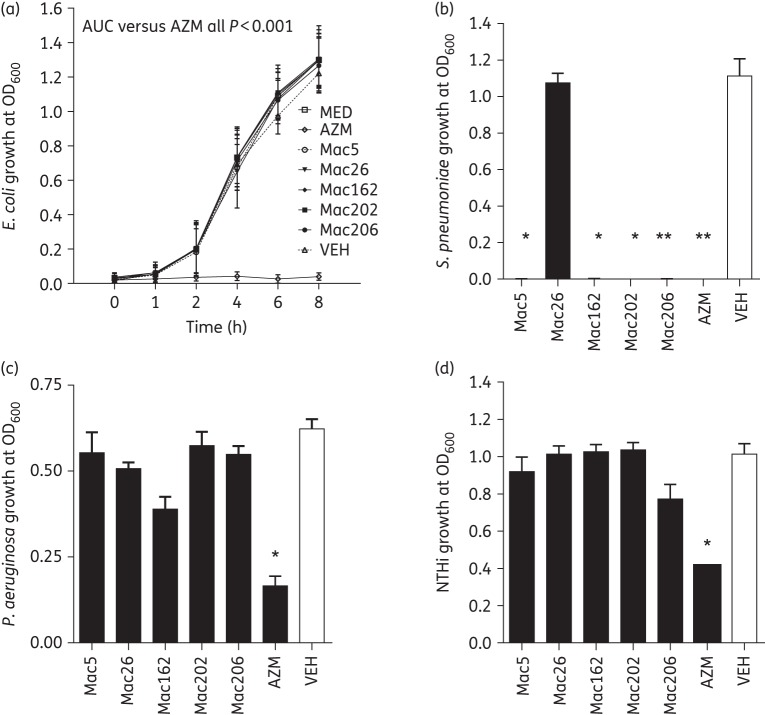
Novel macrolides demonstrated limited antibacterial activity against *E. coli* and respiratory pathogens compared with azithromycin. (a) *E. coli* was grown in the presence or absence of 50 μM azithromycin (AZM) or each novel macrolide (50 μM) for 8 h. AZM inhibited the growth of *E. coli*, while each novel macrolide, vehicle (VEH) and medium treatment (MED) had no effect. *P* < 0.001 for each macrolide, vehicle and medium compared with azithromycin treatment calculated from AUC. *n* = 3. (b) *S. pneumoniae* was grown in the presence or absence of 50 μM AZM or each novel macrolide (50 μM) for 16 h. All novel macrolides except Mac26 inhibited growth compared with vehicle. *n* = 3. (c) *P. aeruginosa* was grown in the presence or absence of 50 μM AZM or each novel macrolide (50 μM) for 4 h. No novel macrolides inhibited growth compared with vehicle; however, AZM showed inhibition. *n* = 3. (d) NTHi was grown in the presence or absence of 50 μM AZM or each novel macrolide (50 μM) for 4 h. No novel macrolides inhibited growth compared with vehicle; however, AZM showed inhibition. *n* = 3. *P < 0.05 and **P < 0.01 versus vehicle-treated cultures.

We also wanted to compare the antibacterial properties of each of the five novel macrolides with azithromycin. We found that none of the five macrolides had antibacterial properties compared with azithromycin using *E. coli* growth as a model (Figure 7a). Mac5 and all other macrolides failed to reduce *E. coli* growth at any timepoint (1–8 h) and all were significantly different from azithromycin by AUC analysis, which totally prevented *E. coli* growth (Figure 7a). However, Mac5, Mac162, Mac202, Mac206 and azithromycin all displayed antibacterial activity against the Gram-positive bacterium *S. pneumoniae* culture at 16 h, while Mac26 did not (Figure 7b). Similar to the findings with *E. coli*, no novel macrolide suppressed growth of the Gram-negative respiratory pathogens *P. aeruginosa* at 4 h (Figure 7c) and NTHi at 4 h (Figure 7d).

### Hit expansion around the Mac5 structure identified several oleandomycin derivatives with IFN-augmenting, anti-inflammatory and antibacterial activity

**Figure 8.**
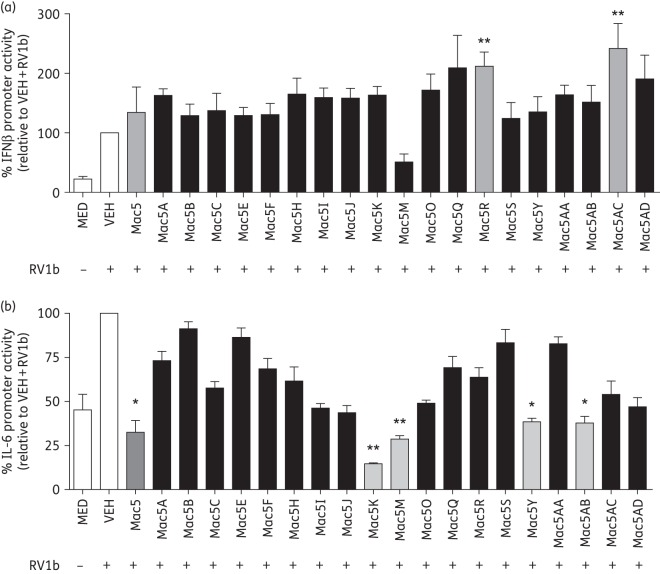
Identification of novel analogues of Mac5 with varying antiviral and anti-inflammatory properties. Thirty analogues of Mac5 were tested for (a) IFNβ promoter-augmenting activity and (b) anti-inflammatory activity by suppression of the IL-6 promoter. *P < 0.05 and **P < 0.01 for each macrolide compared with vehicle (VEH) treatment. Medium (MED)-treated cells are also shown. *n* = 3.

**Figure 9.**
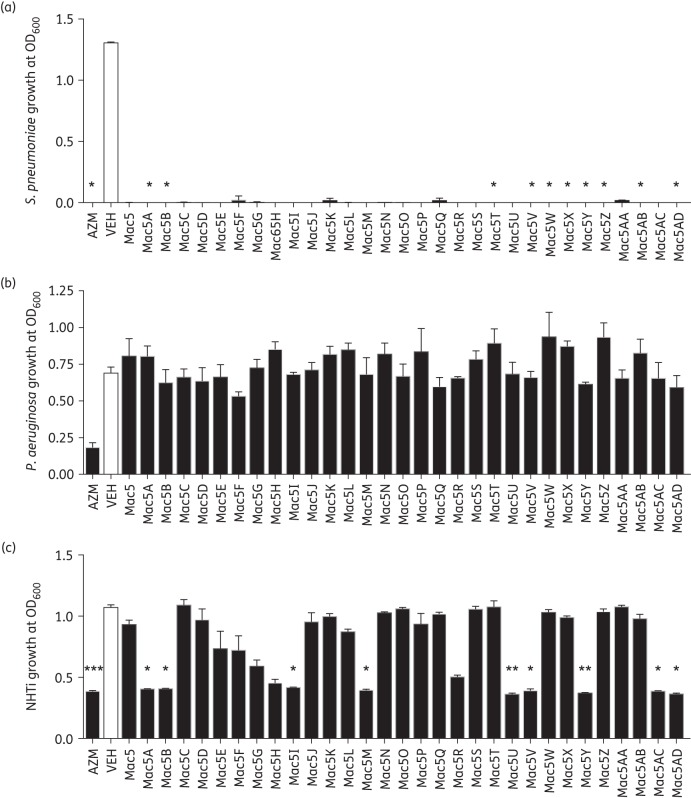
Identification of novel analogues of Mac5 with varying antibacterial properties. Analogues of Mac5 were tested for antibacterial properties against respiratory pathogens *S. pneumoniae*, *P. aeruginosa* and NTHi. (a) All novel Mac5 analogues (50 μM) showed antibacterial properties against *S. pneumoniae* versus vehicle (VEH). Azithromycin (AZM; 50 μM) also showed significant activity. *n* = 3. (b) No Mac5 analogue showed any activity against *P. aeruginosa*. AZM showed a suppressive, but not significant, effect versus VEH. *n* = 3. (c) Some Mac5 analogues showed activity against NTHi. Mac5A, Mac5B, Mac5I, Mac5M, Mac5U, Mac5V, Mac5Y, Mac5AC and Mac5AD all had significant activity versus VEH. AZM also showed significant activity. *n* = 3. *P < 0.05, **P < 0.01 and ***P < 0.001 versus VEH.

A further 30 4″-ureido-oleandomycin derivatives related to Mac5 were screened from the Pfizer compound collection (synthesis of these compounds is described in Patent US4098993^34^). These 30 derivatives of Mac5 were first assessed for IFN-augmenting activity using a reporter system (Figure 8a). We found that only Mac5R and Mac5AC significantly induced IFNβ promoter activity with RV1b relative to vehicle, while several showed non-significant trends for induction. Mac5M appeared to reduce IFNβ reporter activity. Several compounds showed anti-inflammatory activity using the IL-6 promoter reporter (Figure 8b), with the parental Mac5 and derivatives Mac5K, Mac5M, Mac5Y and Mac5AB significantly reducing activity relative to vehicle. Other compounds exhibited non-significant trends for reduction. We next assessed each compound in the antibacterial assays, using *S. pneumoniae*, *P. aeruginosa*, NTHi and *E. coli.* We found that all Mac5 derivatives suppressed *S. pneumoniae* growth, although not all gave significant reductions compared with vehicle (Figure 9a). Results for the Gram-negatives were more variable. Again, no macrolide could suppress growth of *P. aeruginosa* (Figure 9b), while Mac5A, Mac5B, Mac5I, Mac5M, Mac5U, Mac5V, Mac5Y, Mac5AC and Mac5AD all had significant activity against NHTi (Figure 9c). *E. coli* was also examined and Mac5B, Mac5A, Mac5AC and Mac5M significantly inhibited *E. coli* relative to vehicle, while Mac5H, Mac5Y and Mac5AD showed greater suppression that was also significant versus vehicle (Figure S7). All other Mac5 derivatives had no effect on *E. coli* growth (data not shown). Each of the three properties is depicted in Figure S7, with anti-inflammatory activity (IL-6 promoter) and antiviral activity (IFNβ promoter) plotted against each other. Anti-inflammatory and antiviral activity were not significantly related (Spearman correlation *r* = −0.1272, *P* = 0.5825). Cellular toxicity assays using the MTT assay showed that the above macrolides were not toxic in BEAS-2B cells (Figure S8).

### Structural description of Mac5-like oleandomycin derivatives

**Figure 10.**
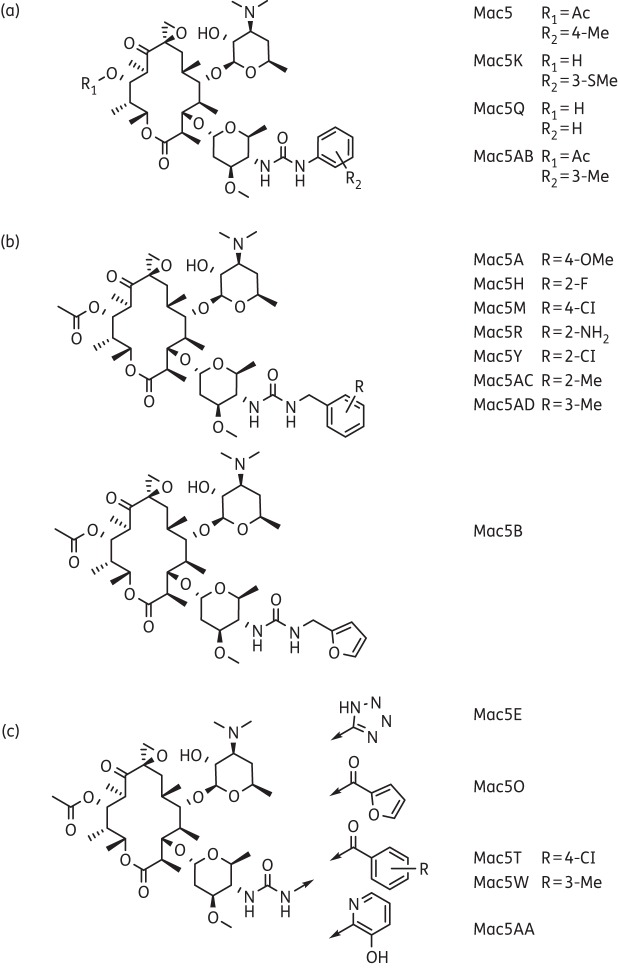
Structures of oleandomycin derivatives with and without antibacterial properties towards Gram-negatives. (a) Phenyl ureas, including Mac5 and derivatives Mac5K, Mac5Q and Mac5AB. (b) Benzyl/methylene heterocyclic ureas, including derivatives Mac5A, Mac5H, Mac5M, Mac5R, Mac5Y, Mac5AC, Mac5AD and Mac5B. (c) Acyl or heterocyclic ureas, including derivatives Mac5E, Mac5O, Mac5T, Mac5W and Mac5AA.

The 30 derivatives differed in substitution of the urea and generally fall within the following classes: (i) direct phenyl ureas; (ii) benzyl/methylene heterocyclic ureas; and (iii) acyl ureas (Figure 10, stereochemistry has been included). One interesting trend is that Mac5, Mac5K, Mac5M, Mac5Y and Mac5AB significantly reduced RV1b-induced IL-6 promoter activation and contain both phenyl and benzyl ureas, but only a subset of the benzyl ureas inhibited the growth of Gram-negative NHTi and *E. coli* compared with vehicle (those being Mac5A, Mac5B, Mac5H, Mac5M, Mac5Y, Mac5AC and Mac5AD). The acyl ureas and directly attached heterocyclic ureas (e.g. Mac5E, Mac5O, Mac5T, Mac5W and Mac5AA) tended to show weaker activity as a class in the assays.

## Discussion

Asthma, COPD and more recently CF are respiratory diseases that are exacerbated by respiratory virus infections.^24,35–39^ The underlying aetiology is complex, but involves overzealous inflammation,^24,37,40–42^ bacterial colonization^38^ and impaired antiviral immunity.^16,24,29^ In this study, we identified several new macrolides with virus-induced IFN-augmenting properties, with additional anti-inflammatory and antibacterial potential. These small molecules may be useful chemical starting points for future drug discovery campaigns for new therapies for asthma, COPD and also CF exacerbations.

One major challenge in drug discovery is selecting a reproducible readout in the screening phase that is biologically relevant to the desired host's response *in vivo*. In the present study, we have combined immortalized cell lines and a luciferase-based screening assay with studies in relevant (asthmatic) disease cells to confirm findings. The ability to firstly measure IFN responses to virus infection by screening a library of molecules coupled with quick validation experiments in primary cells provides an extremely powerful means to identify novel drugs with unique antiviral activity. Models of RV infection in BECs show virus-dependent induction of IFN that correlate with increases in the ISGs MxA and viperin^43–45^ and direct antiviral activity;^16,20^ therefore, detection of IFN production serves as viable surrogate readout of antiviral activity through induction of ISG pathways. Furthermore, Mac5 reduced virus replication, proving antiviral activity for both viruses tested. Mac5 reduced RV release in 7/10 asthmatic BECs tested, confirming that Mac5 does have antiviral potential.

Previously, a promoter-linked bioluminescent detection assay as a method of detecting promoter activity in a high-throughput screen of small molecules was employed as a robust way of analysing a diverse library of compounds in a cost- and time-effective manner.^46^ The use of immortalized cell lines to generate initial data allows a scaling down of experiments to increase throughput in a timely manner. We found using the IFNβ promoter in reporter assays a useful tool to screen macrolides and identified several interesting molecules using this system. We thought it was essential, however, to verify findings seen in immortalized cell lines in a more clinically relevant model such as primary cell culture and made use of both normal and asthmatic BECs to do so. As inherent differences may exist between the two models, confirmatory studies in primary cells are mandatory. This important point is underscored in our study by the fact that despite identifying five macrolides that augmented IFNβ and IFNλ1 promoter activation, only one (Mac5) consistently induced endogenous mRNA in primary BECs. There are many reasons why the reporter-based assay identified so many false positives by analysis of endogenous genes. BEAS-2B cells are an immortalized human bronchiolar cell line and may exhibit subtle differences from normal primary BECs (primary cells from cadavers) in response to both macrolide compounds and virus infection. Additionally, in this study we looked at mRNA expression at 24 h in BECs; due to the transient nature of mRNA within cells, examining mRNA expression at a single timepoint provided only a snapshot of the effects of the novel macrolides. This is kinetically different from the accumulation of stable luciferase protein produced from promoter activation over the course of 24 h (as in phases 1 and 2 of the screen) and thus may explain why the BECs yielded different results to those in the first two phases. Assessing the effects of macrolides on BEC-derived gene transcription over the course of 24 h may confirm previous non-augmenters to indeed have IFN-augmenting ability, but with a more limited duration of action and a peak augmenting effect at a time other than 24 h.

A limited number of studies have also previously identified small molecules that are capable of augmenting host antiviral responses to viruses.^47–49^ In theory, such small molecules capable of boosting the host's antiviral responses to viral infection could prove to be an effective alternative to recombinant IFN therapy in the prophylactic treatment of virally associated asthma or COPD exacerbations.^25^ IFN production in response to viral infection is typically initiated by the activation of host cell PRRs such as Toll-like receptors (TLRs).^43^ Several potent TLR agonists have been reported that have antiviral activity and other immunomodulatory properties.^50^ Previously, R-837 (imiquimod), a TLR7/8 agonist, was shown to be antiviral against herpes simplex virus^47^ and human papilloma virus infection and is currently approved for use as a topical antiviral therapy for genital warts.^48^ We believe that azithromycin and the novel macrolides we have identified such as Mac5 are unlikely to be functioning as TLR agonists. An important difference between macrolides and TLR agonists is that the macrolides did not induce IFN without infection, but rather augmented IFN during infection. While their mechanism(s) of action are unknown, this may give important clues as to their function. It is possible that azithromycin and Mac5 ‘prime’ the IFN gene expression or ISG pathway, such that when infection occurs, a greater magnitude and duration of IFN expression occurs. Further studies, however, are required to understand the mechanism of action of azithromycin and Mac5.

The ability to augment virus-induced IFN rather than spontaneously induce IFN in the absence of infection may also have some clinical advantages. Some trials with IFN as a therapy have resulted in unwanted local inflammation and side effects.^51^ In certain systems, IFN is known to induce inflammation.^52^ As a therapy for inflammatory disorders such as asthma and COPD, creating further inflammation in the absence of infection may be detrimental rather than beneficial. As azithromycin and other macrolides do not have this property, these side effects could in theory be avoided.

Other small molecules are known augmenters of IFN induction. Thomas *et al.*^49^ have shown that ribavirin-treated cells augment ISG responses in a model of hepatitis C virus infection. Furthermore, Patel *et al.*^46^ have described a novel high-throughput screen that identified idarubicin, a small molecule capable of augmenting the IFN signalling pathway *in vitro* leading to increased ISG induction; however, this molecule has yet to be tested *in vivo.* The ability to identify molecules with IFN-augmenting characteristics in a high-throughput, biologically relevant screen and confirm action in primary cells is thus a powerful tool for drug discovery and may provide future novel therapies for the treatment of RV-associated asthma exacerbations as well as other viral infections.

Several macrolides have recently been assessed as a therapy for asthma and COPD exacerbations.^53^ While efficacy has varied possibly due to macrolide-specific effects, patient selection and study design, overall they provide encouraging evidence that macrolides can reduce the frequency, severity or duration of exacerbations. A current scientific debate concerning macrolide therapy is their mechanism of action. As azithromycin and clarithromycin are believed to have antiviral, antibacterial and anti-inflammatory properties, how they control exacerbations is likely complex. Telithromycin has also shown efficacy in controlling asthma exacerbations,^54^ yet has no known antiviral activity.^28^ As the clinical studies conducted with macrolides are not designed to understand mechanisms, it is unclear whether or not they are working via one or more of these properties. A recent concern highlighted by such studies is their associated side effects, particularly with azithromycin,^55^ which may be class specific. While the antibacterial properties of macrolides may in part explain their effectiveness in controlling exacerbations, issues such as long-term use leading to bacterial resistance^56^ or perturbing the natural microbiome^57,58^ have been raised, therefore perhaps contradicting their usefulness as treatments for exacerbations. The discovery of macrolides that do not have broad-spectrum antibacterial properties, but retain antiviral and anti-inflammatory properties, may represent a forward step in the design of next-generation macrolide-based therapies for asthma and COPD exacerbations. Of interest, pandemic influenza infection is also associated with an overabundance of inflammatory cytokine production^59^ and this is associated with disease severity.^60^ Macrolides with anti-inflammatory and IFN-boosting potential may be useful in pandemic influenza infection also.

We examined antibacterial properties of the novel macrolides firstly using *E. coli* as a model organism and then with the important respiratory pathogens *S. pneumoniae*, *P. aeruginosa* and NTHi. Macrolides such as azithromycin promote their antibacterial effects by binding to the large 50S ribosomal subunit in the narrow part of the nascent peptide exit tunnel, thus blocking protein synthesis.^61–63^ For azithromycin and erythromycin, the macrolide ring as well as the sugar moiety (desosamine) at position C5 both make extensive bonds with the *E. coli* peptide exit tunnel.^61,63,64^ We found that Mac26, an azithromycin derivative that had no antibacterial properties in any culture tested including *S. pneumoniae*, has one substitution on desosamine, in that the 2′-OH group has been converted into an acetate and this hydroxyl group is thought to stabilize macrolide–ribosome interactions.^64^ The erythromycin derivatives Mac162, Mac202 and Mac206 also all had no antibacterial effects accept for the Gram-positive *S. pneumoniae* and while other than having slight alterations in stereochemistry, desosamine was chemically the same in these compounds when compared with erythromycin. There were several important substitutions in the 14-membered macrolide ring at positions C10 and C11, however, that could account for a lack of antibacterial effects for Gram-negatives. Less is known about how oleandomycin interacts with bacterial ribosomes. Mac5 differed from oleandomycin by having a hydroxyl group changed into an acetate on the macrocylic ring at C11 and a methyl benzyl urea on the sugar oleandrose. An NMR study of oleandomycin and derivatives thereof showed that positions C10–C12 make extensive interactions with the ribosome;^65^ thus, it is possible that the hydroxyl–acetate substitution at C11 affects this interaction and explains the lack of effect on the Gram-negatives studied.

We further have identified macrolides with (Mac5AC and Mac5AD) and without (Mac5 and Mac5K) antibacterial properties towards Gram-negatives, yet similar antiviral and anti-inflammatory activity. The phenyl ureas Mac5, Mac5K, Mac5Q and Mac5AB all had no antibacterial activity against the Gram-negative bacteria we studied, while all the benzene or methylene heterocyclic ureas with the exception of Mac5R had some level of antibacterial activity. Mac5 having no activity against Gram-negatives and the oleandomycin derivatives Mac5H, Mac5Y and Mac5AD all shared acetates at C11, suggesting that this alone does not account for changes in Gram-negative antibacterial activity. In this report, we offer a brief description of the chemistry of our novel macrolides, but a full investigation into their structure–activity relationship (SAR) was beyond the scope of this study. Further experiments are required to tease out which functional groups are responsible for anti-inflammatory, antibacterial and antiviral activity and, importantly, whether all three functions are due to purely physiochemical properties. These studies are thus among the first to show that these three properties can be selected for in the same molecule and, although a full SAR was not ascertained, we have shown that modest changes in structure can alter the biological properties of the molecule. The macrolides we have identified therefore represent potential useful future alternatives to standard macrolides for asthma, CF and COPD exacerbations. We speculate that during an acute exacerbation, a macrolide with all three properties may be initially beneficial, controlling both viral and bacterial infections and the associated inflammation; however, for longer-term use, a similar macrolide that lacks broad-spectrum antibacterial, but similar antiviral and anti-inflammatory, properties could be administered, subverting bacterial resistance. The future management of asthma, COPD and CF exacerbations may benefit from a selective approach using a set of similar macrolides, but with specific tailored properties.

In summary, we have used a semi-high-throughput reporter-based assay to identify new macrolides with virus-induced IFN-augmenting potential. One macrolide, Mac5, showed increased efficacy and potency compared with a known macrolide, azithromycin. Further studies identified several analogues of Mac5 with various antiviral, anti-inflammatory and even broad-spectrum antibacterial properties. These studies are among the first to highlight the versatility of macrolides and underscore their potential as antiviral, antibacterial and anti-inflammatory molecules. This study has also identified several structures that may serve as chemical starting points for future drug discovery campaigns into new therapies for asthma, COPD and CF exacerbations. Further SAR studies and specific interactions are required to fully understand the multiple biological properties of these molecules.

## Funding

This work was funded by: a BBSRC CASE PhD studentship, with Pfizer as the industrial partner, and an Imperial NIHR BRC Confidence in Concepts Grant (both awarded to M. R. E.); MRC (MRC Centre Grant G1000758); and an Asthma UK Chair to S. L. J. (CH11SJ). S. K. G. was supported by an antimicrobial resistance centre fellowship from Imperial College London. H. G. was supported by an MRC-DTP award to Imperial College London.

## Transparency declarations

This work was funded by a BBSRC CASE PhD studentship, with Pfizer as the industrial partner (awarded to M. R. E.). J. D. P. was employed on the above grant co-funded by Pfizer. M. R. E. has received a speaker's fee from Pfizer. L. R. R., L. H. J. and I. K. are Pfizer employees and shareholders and/or holders of share options. All other authors: none to declare.

### Author contributions

The original concept was conceived by M. R. E., I. K. and S. L. J. All experimental work was performed by J. D. P., J. W. and M. R. E., S. K. G. and H. G. performed the *P. aeruginosa* and NHTi growth curves, under the direction of J. T., and L. R. R. and L. H. J. selected the macrolides and Mac5 derivatives and explored SARs. J. D., E. W. and M. H. A. performed the bronchial brushings of asthmatics, with S. L. J., R. P. W. and M. R. E. responsible for clinical study design. M. R. E. and J. D. P. grew the primary BECs from asthmatics. J. D. P. and M. R. E. analysed the data. J. D. P., M. R. E., L. R. R. and S. L. J. prepared the figures and wrote the paper.

## Supplementary data

Figures S1 to S8 and Tables S1 to S3 are available as Supplementary data at *JAC* Online (http://jac.oxfordjournals.org/).

Supplementary Data
