# Supplementary material for: Identification of novel macrolides with antibacterial, anti-inflammatory and type I and III IFN-augmenting activity in airway epithelium
Source: J Antimicrob Chemother. 2016 Jul 25;71(10):2767–81. doi: 10.1093/jac/dkw222 (PMC5031920; doi:10.1093/jac/dkw222)
Supplement: Supplementary Data [file supp_71_10_2767__index.html]

Identification of novel macrolides with antibacterial, anti-inflammatory and type I and III IFN-augmenting activity in airway epithelium — Identification of novel macrolides with antibacterial, anti-inflammatory and type I and III IFN-augmenting activity in airway epithelium — Supplementary Data 

# Identification of novel macrolides with antibacterial, anti-inflammatory and type I and III IFN-augmenting activity in airway epithelium

## Supplementary Data

Supplementary Data

- Supplementary Data - Docx file
