# Supplementary material for: Identification of novel macrolides with antibacterial, anti-inflammatory and type I and III IFN-augmenting activity in airway epithelium
Source: J Antimicrob Chemother. 2016 Jul 25;71(10):2767–81. doi: 10.1093/jac/dkw222 (PMC5031920; doi:10.1093/jac/dkw222)
Supplement: Supplementary Data [file supp_dkw222_dkw222supp.docx]

**Supplementary data**


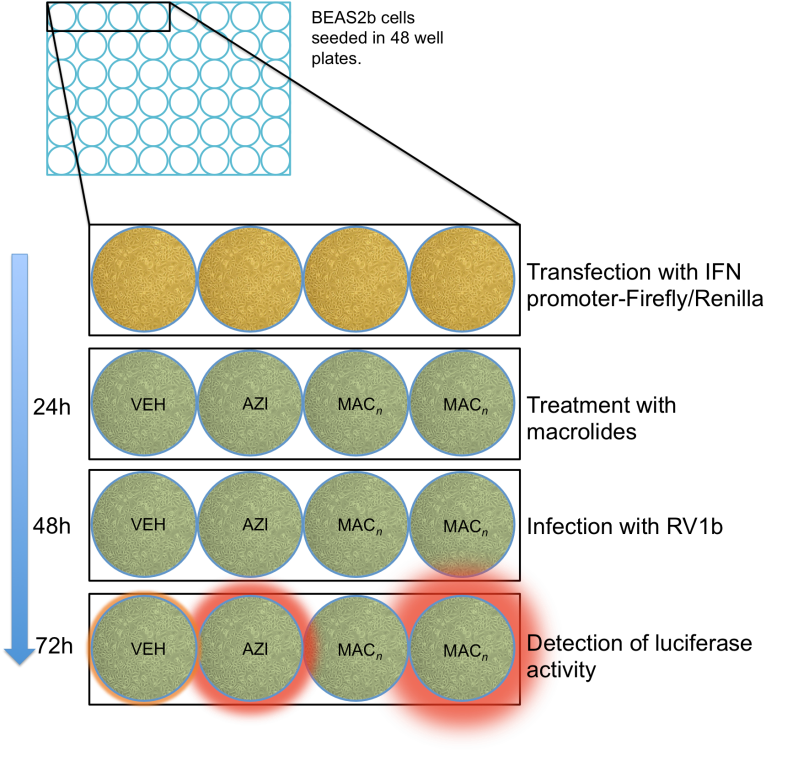
**Fig S1**

Figure S1. Development of a IFNβ promoter luciferase based reporter assay for screening novel macrolides with IFN-augmenting potential. Beas-2B cells were transiently transfected with reporter constructs containing IFNβ (or also IFNλ1 or IL-6) promoter linked to the luciferase (*Firefly*) reporter gene and the transfection control construct *Renilla* luiferase. After 24 hours the cells were treated with novel macrolides for a further 24h. Cultures were then infected with RV1b for 24h before detection of luciferase was assessed to determine promoter activity.

Figure S2. Results of Phase II of the screening process with regard to macrolide class. Beas-2B cells were transiently transfected with reporter constructs containing IFNβ or IFNλ1 promoter linked to the luciferase (*Firefly*) reporter gene and the transfection control construct *Renilla* luiferase. After 24 hours the cells were treated with novel macrolides or vehicle for a further 24h. Cultures were then infected with RV1b for 24h before detection of luciferase was assessed to determine promoter activity. (a) Azithromycin derivatives (AZI-D, *n*=4) and erythromycin derivatives (ERY-D *n*=6) significantly induced the IFNβ promoter versus vehicle (VEH) while the oleandomycin (OLE-D *n*=5) derivatives did not. IFNβ promoter induction by AZI-D, ERY-D and OLE-D were not significantly different from each other. (b) No class of macrolide significantly induced the IFNλ1 promoter. AZI-D, ERY-D and OLE-D were not significantly different from each other. (c) AZI-D and ERY-D significantly induced the combined (sum of) IFNβ and IFNλ1 promoter versus VEH while the OLE-Ds did not. IFNβ promoter induction by AZI-D, ERY-D and OLE-D were not significantly different from each other. **p*<0.05, ***p*<0.01 versus VEH, ns=not significant versus VEH, OLE-D Mac5 is indicated.

Figure S3. Results of MTT assay to assay cell viability on the 5 novel macrolides. Beas-2B cells were treated with 50μM novel macrolides or vehicle, or cell culture medium for 24h. An MTT assay was then employed to determine cell viability, and expressed as OD570. No macrolide gave any difference in cell viability versus vehicle treated cells (all not significant), n=3 experiments.

Figure S4. Mac5 induces MxA and Viperin protein but only during RV infection. BECs were treated with different macrolides for 24h followed by infection with RV1b or medium treatment. Cell lysates were harvested 24h post infection. (a) Effects of each macrolide on RV1b induction of MxA, Viperin and tubulin (load control) expression by western blot. (b) Effects of each macrolide on induction of MxA, Viperin and tubulin (load control) expression by western blot in the absence of infection. Representative picture from 1 experiment.

Figure S5. Dose-response relationship for azithromycin on RV1b induced IFNs and ISGs. BECs were treated with various doses of azithromycin for 24h followed by infection with RV1b. RNA was harvested 24h post infection and converted to cDNA and qPCR performed for (a) IFNβ (b) IFNλ1 (c) IFN-λ2/3 (d) MxA and (e) viperin. All data are expressed as a % of RV1b infected control treated with vehicle n=3 experiments.

Figure S6. Effects of novel macrolides on RV-induced pro-inflammatory cytokine gene expression. BECs were treated with 50μM of azithromycin, or 10 or 50μM of each novel macrolide for 24h followed by infection with RV1b. RNA was harvested 24h post infection and converted to cDNA and qPCR performed for (a) IL-6 and (b) IL-8. All data are expressed as a % of RV1b infected control treated with vehicle n=4-6 experiments.

**Figure S7. Mac5 analogues showed some suppressive activity against *E. coli.*** a) 50μM Mac5 or Mac5A, Mac5B, Mac5H, Mac5M, Mac5Y Mac5AB and Mac5AC inhibited the growth of *E. coli* compared to Vehicle (VEH) treatment. Medium (MED) treated cells are also shown. Azithromycin (AZI) completely inhibited *E. coli* growth. b) Graphic comparison of **novel analogues of Mac5 showed varying anti-viral, anti-bacterial (*E. coli*) and anti-inflammatory properties.** Several compounds inhibited the growth of *E. coli*, which appeared to not be related to either IL-6 suppression or IFNβ induction.

**Figure S8. Results of MTT assay to assay cell viability on 30 Mac5 analogues.** Beas-2B cells were treated with 50μM novel macrolides or vehicle, or cell culture medium for 24h. An MTT assay was then employed to determine cell viability, and expressed as OD570. Only Mac5T, Mac5U, Mac5V and Mac5W gave significant reductions in cell viability and were considered to be toxic ***p<0.001 versus vehicle (VEH) treated cells. medium treated cells are also shown *n*=3 experiments.

**Table S1. Design and results of a multi-phase screening regime that identified novel macrolides with IFN augmenting activity.**

| **Phase** | **No. Macrolides** | **Cell type** | **Read-out** | **Notes** |
| --- | --- | --- | --- | --- |
| 1. | 226 | BEAS-2B | IFNβ-Luc reporter | Compounds that alter cell morphology/ disrupt the monolayer were excluded.  Compounds that augment IFNβ-Luc activity ≥1.5 fold mean +3SD above AZI were progressed to Phase 2. |
| 2. | 15 | BEAS-2B | IFNβ-Luc, IFNλ1-Luc reporter | Compounds that significantly augmented IFNβ-Luc or IFNλ-Luc activity were progressed to Phase 3. |
| 3. | 5 | HBEC | IFNβ, IL-28, IL-29, MxA, Viperin, IL-6 and IL-8 mRNA expression. | One “lead” compound was chosen and assessed for SAR. |

Table S2. Comparison between Mac5 and azithromycin in IFN-agumenting ability and suppressing RV replication in HBECs.

| Assay | Azithromycin  **(% mean ± SEM)^†^** | Mac5  (% mean ± SEM)^†^ | ***p* value^‡^** | **Fold difference^§^** |
| --- | --- | --- | --- | --- |
|  |  |  |  |  |
| IFNβ reporter  IFNλ1 reporter | 216 ± 18.5  220 ± 51.2 | 279 ± 18.2  199 ± 26.5 | 0.07  0.73 | 1.29  0.90 |
| IFN mRNA  IFNβ  IFNλ1  IFNλ2/3 | 511 ± 131  557 ± 175  674 ± 232 | 1,193 ± 459  1,898 ± 742  1,882 ± 782 | 0.18  0.10  0.16 | 2.33  3.41  2.79 |
| ISG mRNA  Viperin  MxA | 484 ± 191  252 ± 37.6 | 2,808 ± 1641  539 ± 304 | 0.18  0.36 | 5.80  2.13 |
| RV1B release | 50.0 ± 9.90 | 35.8 ± 9.22 | 0.33 | 0.72 |

^†^Expressed as a % of cells treated with vehicle and infected with RV1b.

^‡^Compares cells treated with azithromycin (50μM) and infected with RV1b versus MAC5 (50μM) treated and RV1B infected cells.

^§^Data from MAC5 treated RV1b cells divided by azithromycin treated, RV1b infected cells.

**Table S3. Asthmatic patient Data**

|  | |
| --- | --- |
| **Characteristic** | **Median (range)** |
| *n* | 10 |
| Age | 29.50 (23-39) |
| Sex (% male) | 50.0 |
| FEV1 (%) | 85.5 (63-100) |
| IgE | 74.35 (25-800) |
| No. of +ve SPT | 2 (1-6) |
| PC_20_ | 1.00 (0.03-8) |
| ACQ | 1.50 (1-4.5) |
|  |  |
|  |  |
